# The Energy Storage Properties of Refrigerants (R170, R134a, R143a, and R152a) in Mof-5 Nanoparticles: A Molecular Simulation Approach

**DOI:** 10.3390/ma12213577

**Published:** 2019-10-31

**Authors:** Qiang Wang, Zhengyong Huang, Shucheng Ou, Ruiqiang Zhang

**Affiliations:** 1 State Key Laboratory of Power Transmission Equipment & System Security and New Technology School of Electrical Engineering, Chongqing University, Chongqing 400044, China; wangqiang870609@163.com; 2 Baotou Power Supply Bureau of Inner Mongolia Electric Power Group Co., Ltd., Baotou 014000, China; yangcheng136804@vip.sina.com (S.O.); zrq102@126.com (R.Z.)

**Keywords:** refrigerant, MOF-5, adsorption, energy storage, molecular simulation

## Abstract

The thermophysical properties of refrigerant can be modified via adding solid materials to it. In this paper, molecular simulations and thermodynamic calculations were employed to investigate the adsorption and energy storage of ethane (R170), 1,1,1,2-tetrafluoroethane (R134a), 1,1,1-trifluoroethane (R143a), and 1,1-difluoroethane (R152a) in metal organic framework (MOF)-5 nanoparticles. The results show that the fluorine atom in the refrigerants will strengthen the adsorption of refrigerants in MOF-5. However, the fluorine-free refrigerant, R170, owns larger enthalpy difference of desorption than the other refrigerants with fluorine under high pressure. The thermal energy storage capacity of the refrigerant/MOF-5 mixture is larger than that of the pure refrigerant at low pressure. Also, the negative enhancement of the energy storage property of the mixture is found in some cases when the refrigerant experiences phase transition.

## 1. Introduction

The rapid development of modern society comes at the expense of massive energy consumption and environmental degradation. Therefore, various approaches have been proposed to achieve sustainable development. Modifying the thermophysical properties of working fluid to enhance the efficiency of the thermodynamic cycle is one of the most effective methods to save energy and reduce emissions [1,2,3,4]. Actually, the thermal conductivity of working fluid can be enhanced by mixing with nanoparticles, which have been extensively studied in the past decades [5,6,7,8,9].

In addition, nanofluids with nanoporous materials have the potential to store energy, which is based on the exothermic adsorption and endothermic desorption of fluid into nanopores [10]. Xu et al. [11] studied the energy capture mechanism of water-based nanofluid. The results showed that nanofluid systems have high energy density. McGrail et al. [12,13] investigated the energy storage property of metal-organic heat carrier nanofluids (MOHCs), which consist of refrigerants mixed with metal organic frameworks (MOFs). Since refrigerants are widely used in refrigeration, heat pump and organic Rankine cycles (ORCs) and MOHCs have limitless applications in the energy industry [14,15].

MOFs, which possess high specific surface area for adsorption and separation, are coordination networks with organic ligands containing potential voids [16,17]. Henninger et al. [18] synthesized a water-stable MOF for refrigeration, heat pump, and thermal storage. Rezk et al. [19] studied the adsorption properties of ethanol in six kinds of MOFs for cooling. Zheng et al. [20] reported the adsorption isotherms of 1,1,1,2-tetrafluoroethane (R134a) in Ni-MOF-74 by experimentation. Elsayed et al. [21] experimentally investigated the characteristics of CPO-27(Ni) MOF and proved its feasibility for energy storage. The adsorption and energy storage properties of refrigerants in MOFs should be further studied for the various refrigerants and MOF structures [22,23,24,25].

Since the adsorption and energy conversion of refrigerant molecules and MOF structure interact at nanoscale, molecular simulation (MS) [26,27,28,29] is an ideal approach to investigate the energy storage mechanisms of MOHCs. Getman et al. [30] reviewed the reports of MS of CH_4_, H_2_, and C_2_H_2_ storage in MOFs and found that MS is a powerful tool for understanding gas adsorption in MOFs. Sun et al. [31] studied the structural, diffusive, and adsorption properties of hydrocarbons (n-hexane and cyclohexane) in a nickel-based MOF (Ni\DOBDC) by molecular dynamics (MD) simulations. The binding of n-hexane in Ni\DOBDC is stronger than that of cyclohexane. Kong et al. [32] analyzed the functional groups in MOFs with experimental measurements and MS. Recently, we have analyzed the thermal storage properties of the CO_2_/MOF-5 mixture with MD and grand canonical Monte Carlo (GCMC) simulations [33]. The results showed that the energy storage capacity is affected by the mass ratio of MOFs and pressure.

Among the various MOF structures, MOF-5 is a classic three-dimensional skeleton composed of Zn^2+^ and terephthalic acid with a specific surface area of up to 2000 m^2^/g [34]. Ethane (R170), 1,1,1,2-tetrafluoroethane (R134a), 1,1,1-trifluoroethane (R143a) and 1,1-difluoroethane (R152a) are the ethane-like refrigerants that are widely used at present [1]. Here, the adsorption and energy storage of the above four refrigerants in MOF-5 nanoparticles are investigated by MS to explore the impact of the refrigerant’s structure on the properties of MOHCs. This work is also expected to provide useful insights on adsorption refrigeration and heat pump cycles [35].

## 2. Method and Computational Details

### 2.1. Thermodynamic Calculation Model

The thermodynamic model for calculating the thermal energy storage capacity of MOHCs (∆h_MOHCs_) when the temperature changes from T_0_ to T_1_ is [12,33]:

(1) $$\Delta h_{\mathrm{MOHCs}}=(1-x)\cdot \Delta h_{\mathrm{Fluid}}+x\cdot (\int_{T_{0}}^{T_{1}}C_{p}\mathrm{dT}{)}_{\mathrm{MOFs}}+x\cdot \Delta h_{\mathrm{desorption}}$$

where ∆h_Fluid_ is the enthalpy change of the pure working fluid, ($\int_{T_{0}}^{T_{1}}C_{p}\mathrm{dT}$)_MOFs_ is the thermodynamic energy change of the MOF nanoparticles, ∆h_desorption_ is the desorption heat of fluid in the MOF nanoparticles, and x is the mass fraction of MOF nanoparticles in MOHCs. Actually, the ∆h_Fluid_ can be conveniently calculated for the thermophysical properties of pure fluid, which have been extensively studied. However, unlike the easy calculation of ∆h_Fluid_, the ($\int_{T_{0}}^{T_{1}}C_{p}\mathrm{dT}$)_MOFs_ and ∆h_desorption_ in MOHCs need further study for the various structures and components of MOFs. Thus, we propose a method to evaluate the ∆h_MOHCs_ [33,36]. More specifically, the enthalpies of the refrigerants are obtained from the National Institute of Standards and Technology (NIST) [37] for computing ∆h_Fluid_. The MD is employed to predict the ($\int_{T_{0}}^{T_{1}}C_{p}\mathrm{dT}$)_MOFs_, and GCMC is used to calculate the ∆h_desorption_.

### 2.2. Simulation Models

**Figure 1:**
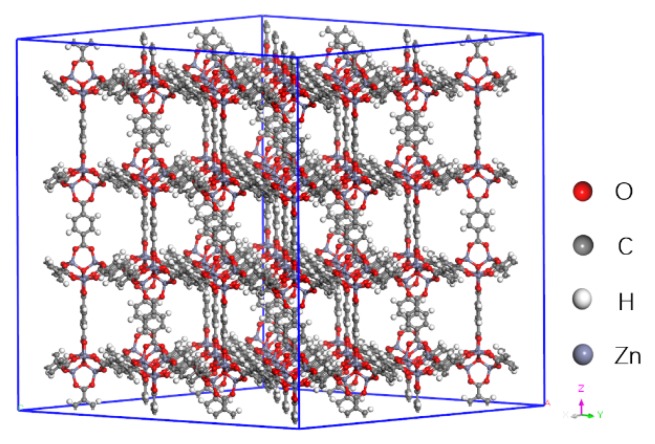
The computational model of metal organic framework (MOF)-5 (2 × 2 × 2 super cell).

**Figure 2:**
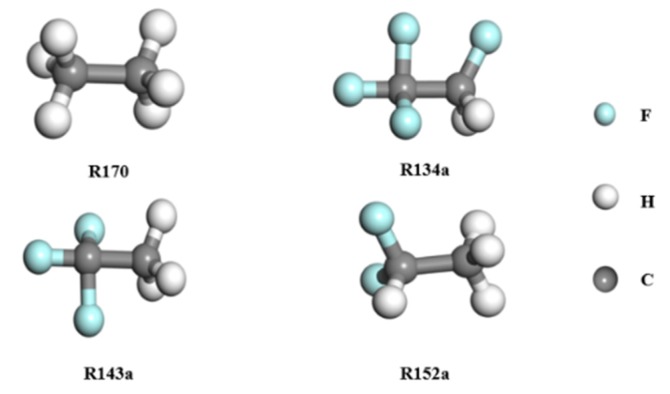
The molecular models of the four refrigerants.

The computational model of MOF-5 is shown in Figure 1 (X: 51.788 Å, Y: 51.788 Å, Z: 51.788 Å), and contains 3392 atoms (including 1536 C, 768 H, 832 O, and 256 Zn). The structures of the studied refrigerants are presented in Figure 2.

The MD and GCMC simulations were performed by the Materials Studio [38]. The COMPASS (An ab Initio Force-Field Optimized for Condensed-Phase Applications) force field [39] was employed to describe the interactions of atoms in the system. The long-range Coulombic interactions were solved by the Ewald method [40]. Periodic boundary conditions were applied in X, Y, and Z directions. 

### 2.3. MD Simulation Details

MD simulations were performed in the Forcite module of the Materials Studio. The timestep was set at 1 fs and the system was equilibrated at 200 ps to calculate the thermodynamic energies of MOF-5 from 300 K to 420 K with intervals of 20 K. The simulations were computed in the NVT (canonical) ensemble. The temperature was controlled by the Berendsen method [41]. The velocity-Verlet algorithm was used to solve the equations of atomic motion. The thermodynamic energy change at different temperatures was the ($\int_{T_{0}}^{T_{1}}C_{p}\mathrm{dT}$)_MOFs_.

### 2.4. GCMC Simulation Details

GCMC simulations were performed in the Sorption module of the Materials Studio. With consideration of the working conditions of refrigeration, heat pump, and ORC [42,43], the adsorption isotherms (300 K, 320 K, 340 K, 360 K, 380 K, 400 K, and 420 K) of the four refrigerants in the MOF-5 structure (Figure 1) was calculated from 1 to 4000 kPa. The fugacity was calculated by the Peng–Robinson equation. For each point of adsorption isotherm, the equilibration time was 100,000 cycles, with another 200,000 cycles for statistic. The desorption heat (∆h_desorption_) was obtained by calculating the adsorption heat at different temperatures. More details of the simulations are described elsewhere [33,36,44].

## 3. Results and Discussion

### 3.1. Adsorption Isotherms

**Figure 3:**
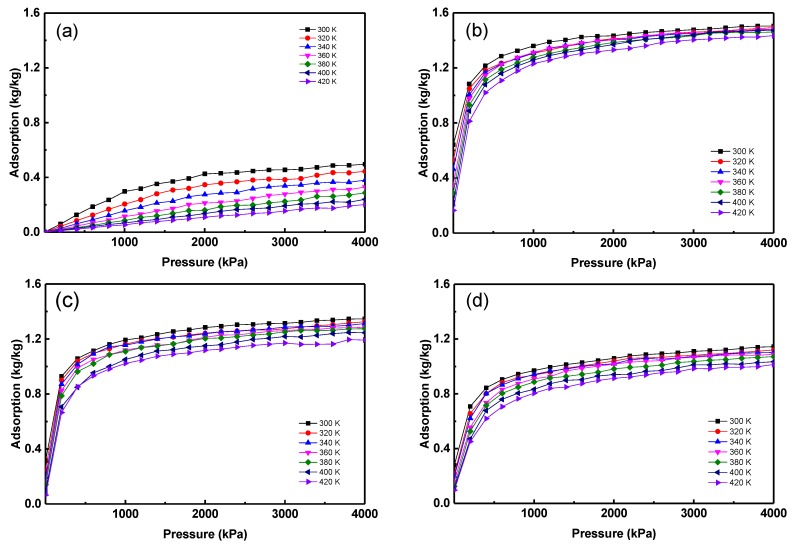
Adsorption isotherms of refrigerants in MOF-5. (**a**) R170, (**b**) R134a, (**c**) R143a, (**d**) R152a.

The adsorption isotherms of the studied refrigerants in MOF-5 are plotted in Figure 3. Since the specific parameters are widely used in thermodynamics, the unit of adsorption is ‘kg/kg’ in this work, which means the mass of refrigerant adsorbed in MOFs at different pressures and temperatures. Apparently, the adsorption of all the refrigerants in MOF-5 increases with the increase of pressure and decreases with the rise of temperature. Generally, the adsorption isotherms of refrigerants in MOFs are the Langmuir isotherms [13,33,36]. However, the adsorption of R170 experiences the initial stage of the Langmuir isotherms, because the investigated pressure is much lower than the saturated pressure of adsorption. Thus, the adsorption of R170 is the lowest among the four refrigerants. Also, the saturated adsorption of the other three refrigerants ranked in ascending order are R152a < R143a < R134a. It should be noted that R170 is ethane (C_2_H_6_), Figure 2, and the structure of the other three refrigerants are similar to ethane. It can be concluded that the replaced fluorine atom in ethane-like structures will increase the adsorption of refrigerants in MOF-5. One of the reasons for this is that the attractive interactions between fluorine and the MOF-5 structure is larger than that between hydrogen and MOF-5 [36]. 

### 3.2. Desorption Heat

**Figure 4:**
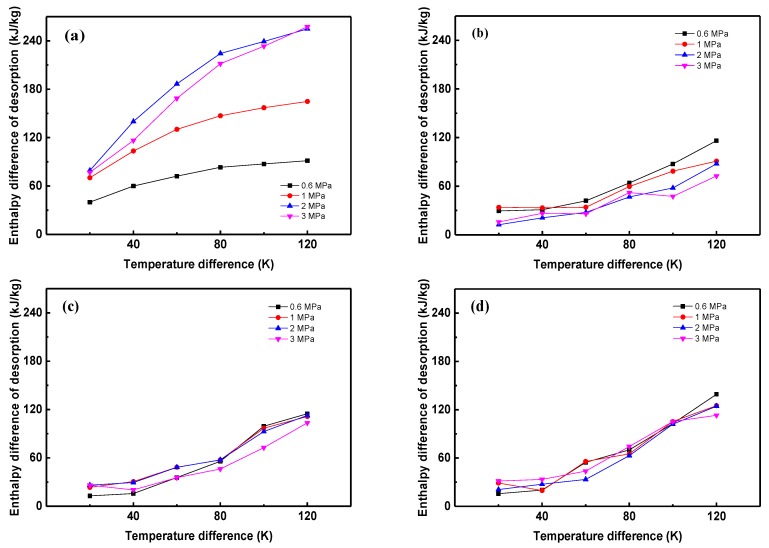
Enthalpy difference of desorption of refrigerants in MOF-5. (**a**) R170, (**b**) R134a, (**c**) R143a, (**d**) R152a.

Since the energy conversion of MOHCs occurs at different temperatures and pressures, the enthalpy difference of desorption at different temperatures are calculated and presented in Figure 4. The reference temperature is set as 300 K. There is a tendency that the enthalpy difference of desorption of refrigerants in MOF-5 increases with the enlargement of the temperature difference. The enthalpy difference of desorption of R170 at 1 MPa, 2 MPa, and 3 MPa are much larger than that of R134a, R143a, and R152a. The reason for this is that the adsorption of R170 notably changes as the temperature changes when the pressure is over 1 MPa, as shown in Figure 3. Meanwhile, the adsorption of R134a, R143a, and R152a fluctuates in a small range at the same status. The fluctuation also leads to the pressure’s unclear impact on the enthalpy difference of desorption.

### 3.3. Energy Storage Property

**Figure 5:**
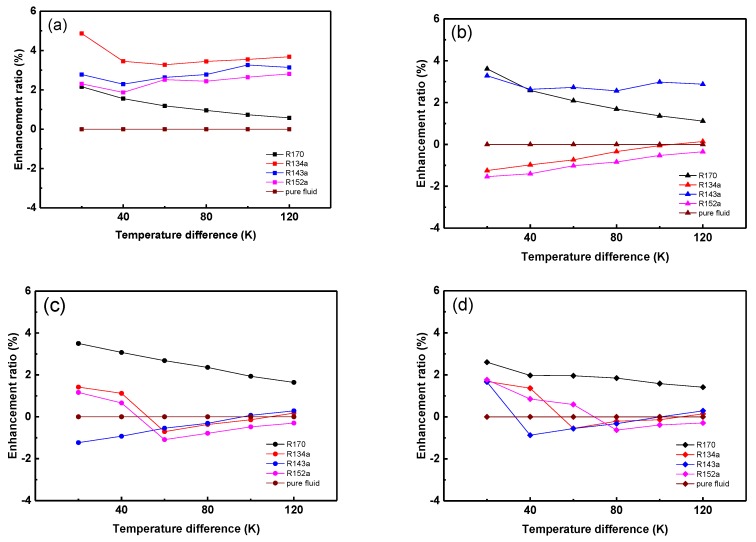
The relationship between the enhancement ratio of the thermal energy change of MOHCs (wt 2%) and temperature difference. (**a**) 0.6 MPa, (**b**) 1 MPa, (**c**) 2 MPa, (**d**) 3 MPa.

Since the mass fraction’s impact on the energy storage property has been discussed in our previous works [33,36], the energy storage capacity of MOHCs with 2% mass fraction MOF-5 has been calculated according to Equation (1). The Cp of MOF-5 is adopted from our previous work [33]. The enthalpy difference of R170, R134a, R143a, and R152a at the appropriate state are shown in the Appendix A. The relationship between the enhancement ratio of the thermal energy change of MOHCs and temperature difference are shown as Figure 5. The MOHCs can store more energy than the pure refrigerant at 0.6 MPa. However, some of the MOHCs store less energy than the pure refrigerant at 1 MPa, 2 MPa, and 3 MPa. The transformation of Equation (1) is

(2) $$\Delta h_{\mathrm{MOHCs}}=\Delta h_{\mathrm{Fluid}}+x\cdot ((\int_{T_{0}}^{T_{1}}C_{p}\mathrm{dT}{)}_{\mathrm{MOFs}}+\Delta h_{\mathrm{desorption}}-\Delta h_{\mathrm{Fluid}}).$$

**Figure A1:**
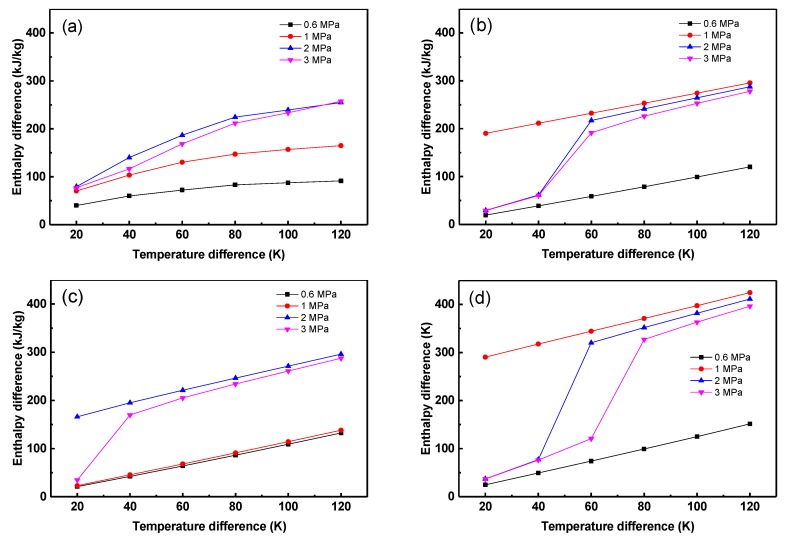
The enthalpy difference of pure refrigerants. (**a**) R170, (**b**) R134a, (**c**) R143a, (**d**) R152a.

It can be inferred that MOHCs definitely strengthen the energy storage capacity of basic fluid when the sum of ($\int_{T_{0}}^{T_{1}}C_{p}\mathrm{dT}$)_MOFs_ and ∆h_desorption_ are larger than ∆h_Fluid_, and vice versa. Since the ∆h_Fluid_ is large when the pure fluid experiences the phase transition, the sharp increase of enthalpy difference in Figure A1 and the negative enhancement of thermal energy storage capacity of MOHCs are usually found in the vicinity of the critical region [36]. Furthermore, the R170/MOF-5 mixture stores more energy than pure R170 in all studied conditions. This is because the R170 keeps in the gas phase under the studied conditions. Another reason is that the enthalpy difference of desorption of R170 in MOF-5 is much larger than that of the other three refrigerants, as discussed in Figure 4.

## 4. Conclusions

The adsorption and energy storage properties of R170, R134a, R143a, and R152a in MOF-5 are investigated in the present paper. The adsorption of R134a is the highest in MOF-5, followed by R143a, R152a, and R170. R170 does not reach the saturated adsorption under the studied conditions. This is because the fluorine in the refrigerants can enhance the attractive interaction between the refrigerant molecules and the MOF-5 structure. However, the enthalpy difference of desorption of R170 is larger than the other refrigerants over 1 MPa. The reason for this is that non-saturation adsorption of R170 leads to the adsorption difference of R170 at different temperature, which is larger than that of R134a, R143a, and R152a at high pressure. The addition of MOF-5 nanoparticles can enhance the energy storage capacity of the studied MOHCs at low pressure (0.6 MPa). Negative enhancement of the energy storage properties of MOHCs are found in some cases, because the enthalpy difference of the pure refrigerant changes dramatically near the phase transition area.

## Appendix A

The enthalpy difference of pure refrigerants.
